# Supplementary material for: MinD2 modulates cell shape and motility in the archaeon Haloferax volcanii
Source: Front Microbiol. 2024 Nov 12;15:1474570. doi: 10.3389/fmicb.2024.1474570 (PMC11588486; doi:10.3389/fmicb.2024.1474570)
Supplement: Supplementary file 1 [file Data_Sheet_1.docx]

**Supplementary materials to**

**MinD2 modulates cell shape and motility in the archaeon *Haloferax volcanii***

**Megha Patro^1,2,+^, Felix Grünberger^3^, Shamphavi Sivabalasarma^1,2^, Sabrina Gfrerer^1^, Marta Rodriguez-Franco^4^, Phillip Nußbaum^1^, Dina Grohmann^3^, Solenne Ithurbide^1,*^ and Sonja-Verena Albers^1,5^**

^1^ Molecular Biology of Archaea, Institute of Biology, Faculty of Biology, University of Freiburg, Freiburg, Germany

^2^ Spemann Graduate School of Biology and Medicine, University of Freiburg, Freiburg, Germany

^3^ Institute of Biochemistry, Genetics and Microbiology, Institute of Microbiology and Archaea Centre, Single-Molecule Biochemistry Lab & Biochemistry Centre Regensburg, University of Regensburg, Universitätsstraße 31, 93053 Regensburg, Germany

^4^Cell Biology, Institute of Biology, Faculty of Biology, University of Freiburg, Schänzlestraße 1, 79104 Freiburg, Germany

^5^ Signalling Research Centres BIOSS and CIBSS, University of Freiburg, Freiburg, Germany

^+^Present address : Structural and Computational Biology Unit, European Molecular Biology Laboratory, Heidelberg, Germany

^*^ Present address: Department de Microbiologie, Infectiologie et Immuunologie, Université de Montréal, Montréal, Québec, Canada

Corresponding Author: sonja.albers@biologie.uni-freiburg.de

**
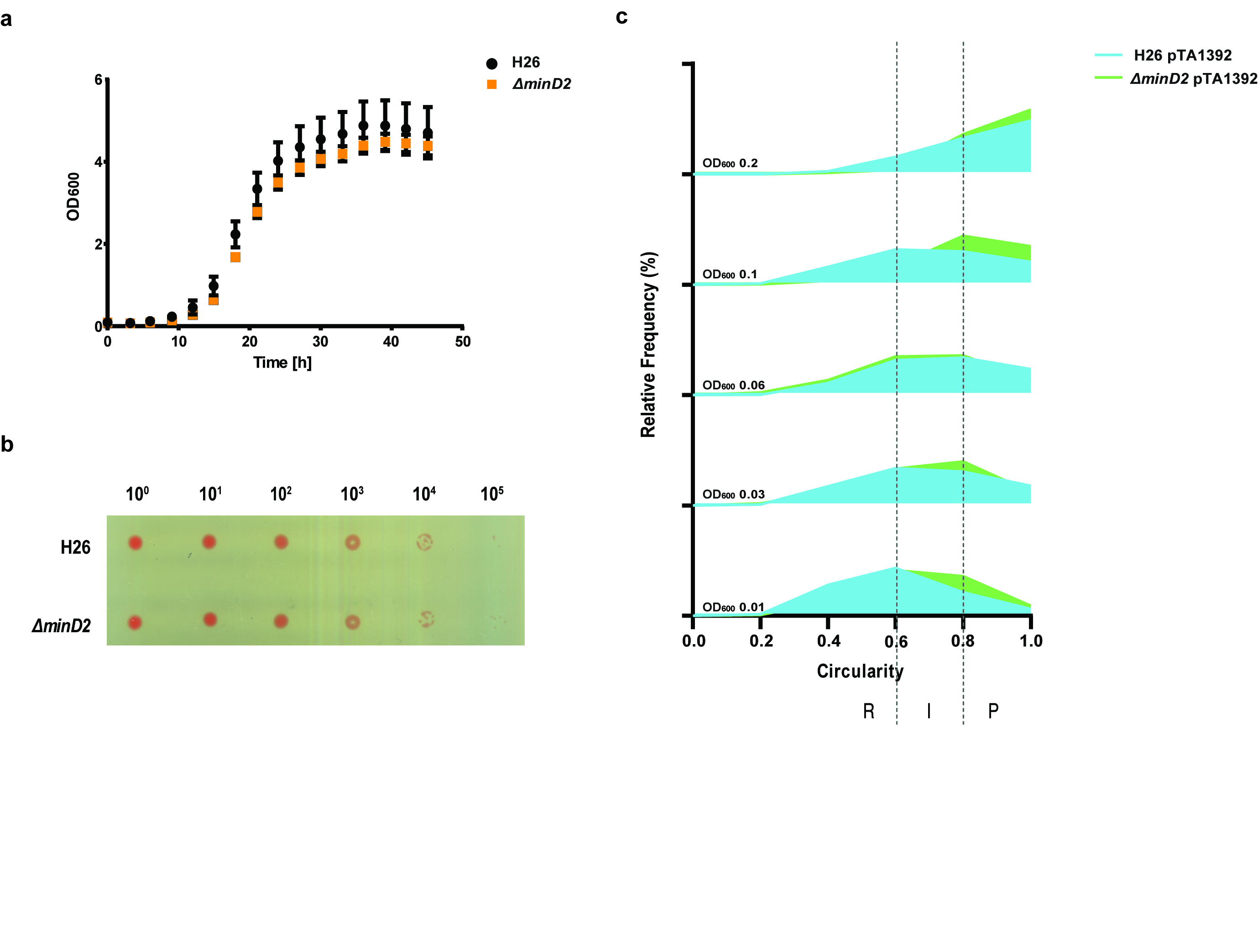
**

**Supplementary figure 1:** (**a**) Growth curve measurement of H26 and *ΔminD2* shows no growth defect in the mutants. (**b**) Spot dilution assay of H26 and *ΔminD2* (+ pTA13929) showing same viability of the mutant *ΔminD2* with respect to wild type H26. **(c)** Relative Frequency measurement of cell circularity of H26 and *ΔminD2* in the presence of plasmid pTA1392. Vertical dashed line represents the different cell type R: Rods, I: Intermediates and P: Plates. Sum of the graph height per OD_600_ equals 100% and Y-axis indicates the percentage of cell population per cell type


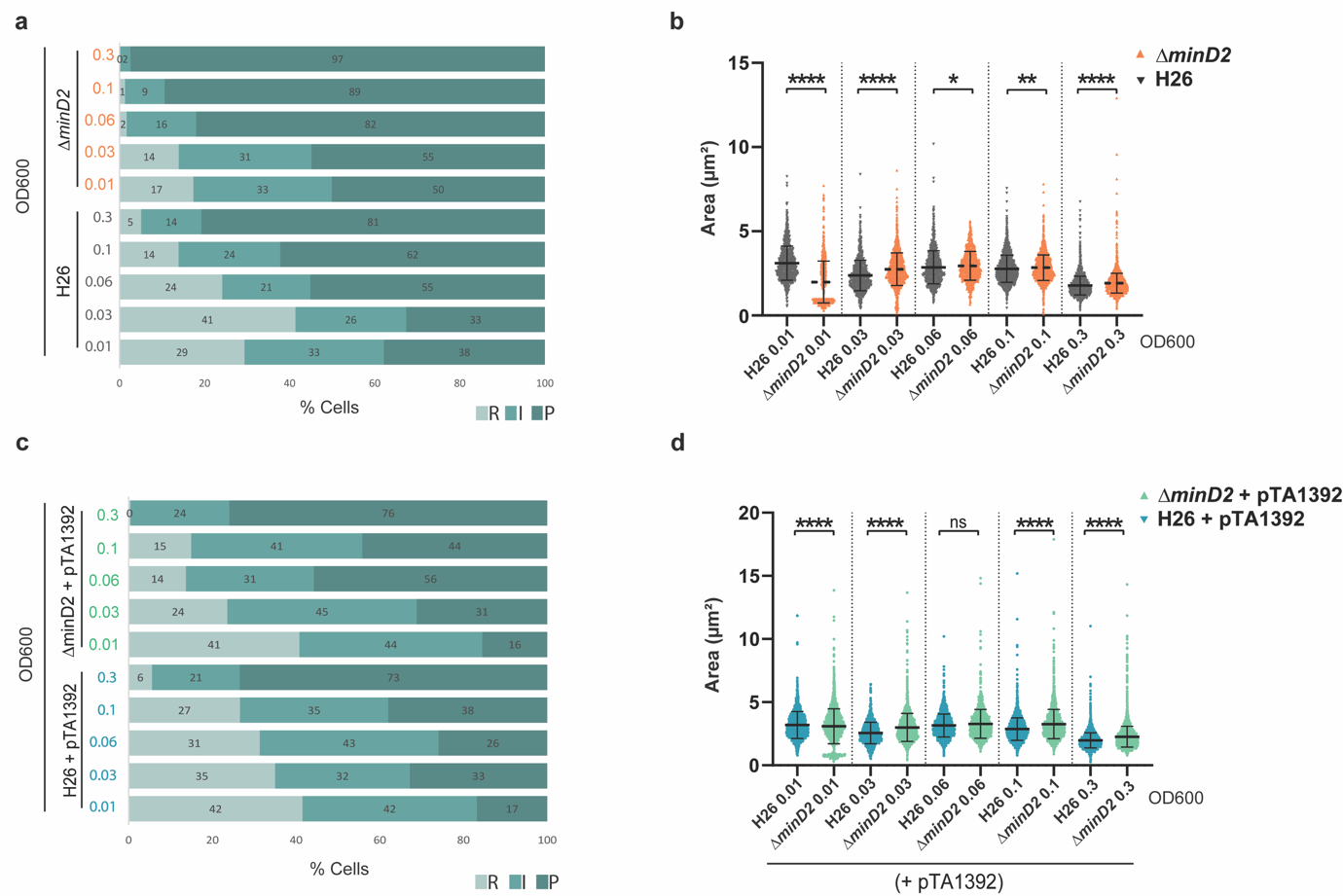


**Supplementary figure 2: Morphological analysis of strains and plasmid**. Bar graph showing the percentage cell present in each cell type (Rods (R), Intermediates (I) and Plates(P)) for (**a**) H26 (grey) and *ΔminD2* (orange); and (**c**) H26 +pTA1392 (blue) and *ΔminD2* + pTA1392 (green). (**b, d**) Scatter plot distribution of cell area (μm^2^) at different OD_600_. n_H26_ >1363 n *_ΔminD2_* > 2769. Calculations were made using 3 independent experiments including more than 3 biological replicates each.


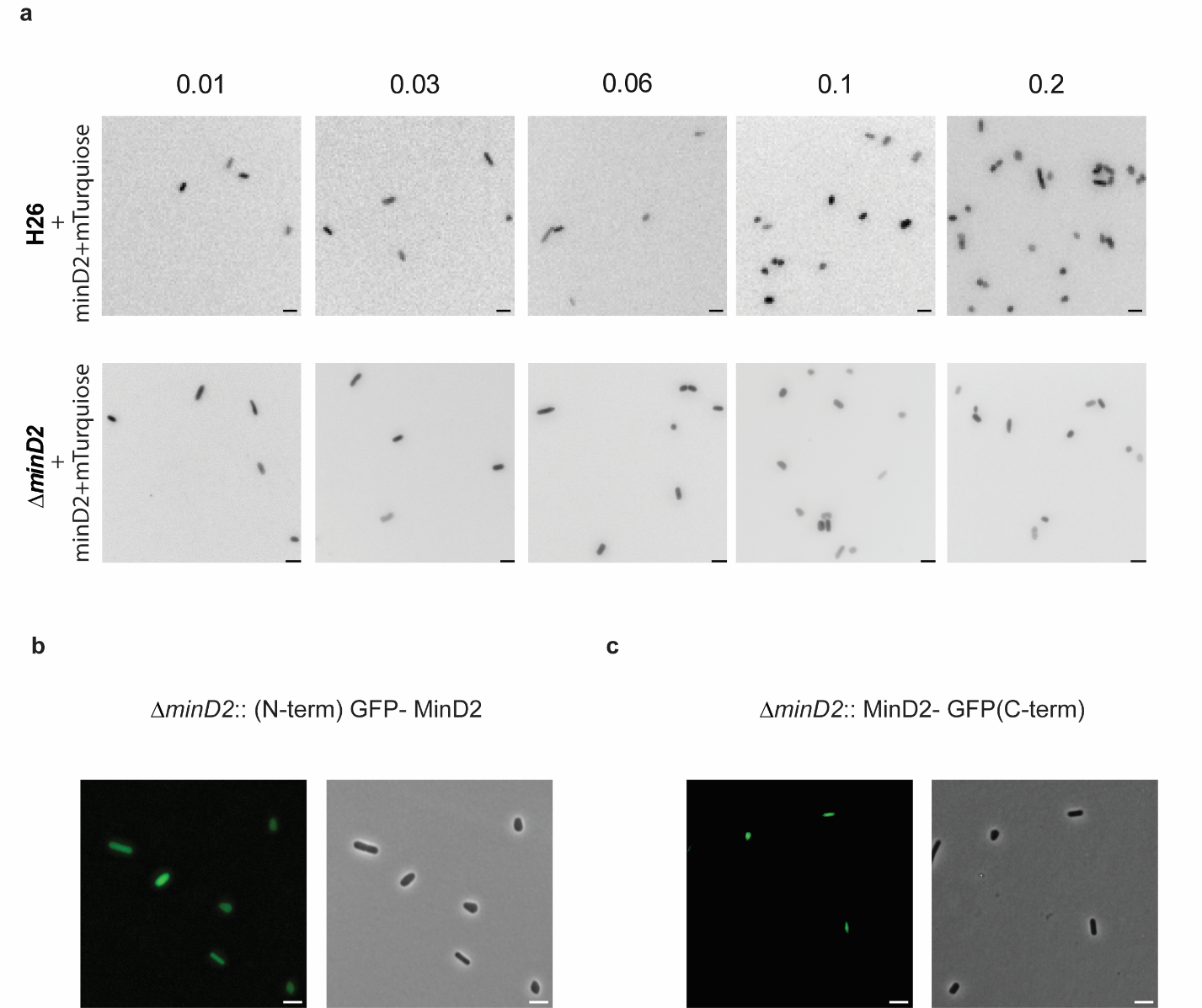


**Supplementary figure 3: Localisation of MinD2 shows diffused fluorescence** (**a**) MinD2 + semi flexible linker + mTurquoise showing diffused fluorescence in both wild type (top panel) and *ΔminD2*(bottom panel). Diffused GFP localisation for (**b**) N-term tagged GFP MinD2 and (**c**) C-term tagged MinD2 GFP; Insert are taken at OD 0.06. Scale bar = 4µm


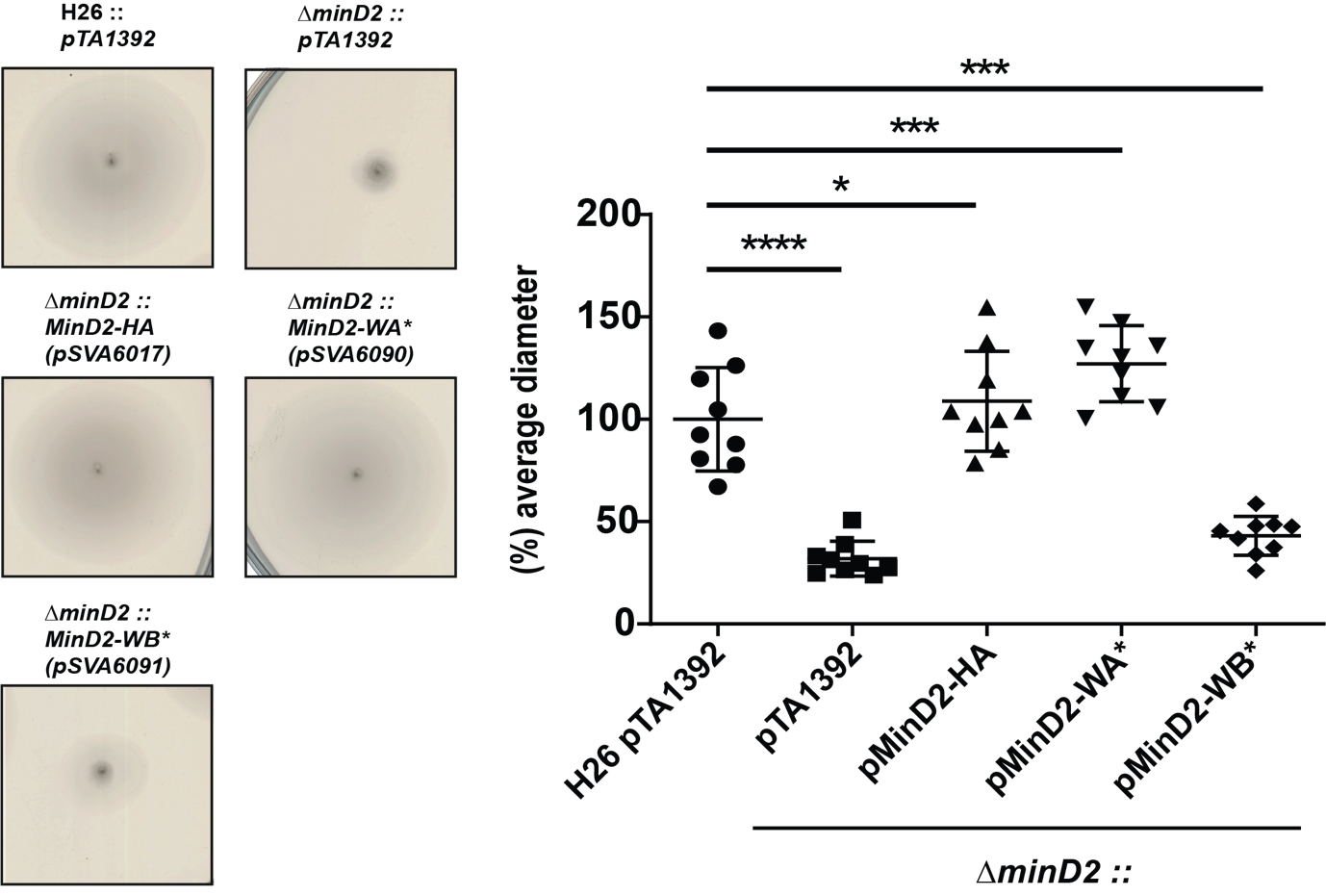


**Supplementary figure 4 Complementation of WA* and WB* in MinD2** (**a**) Motility assays to test swimming activity of WA and WB mutant in *ΔminD2* strain. pMinD2-HA was used as positive control as it was shown to complement full motility function in *ΔminD2* strain.

**Table 1: Strains used in this study**

| **Strain name** | **Genotype** | **Reference** |
| --- | --- | --- |
| ***H.volcanii*** |  |  |
| H26 | *∆pyrE2* | (Allers et al., 2004) |
| HTQ19 | *∆pyrE2∆flaD1* | (Li et al., 2019) |
| HTQ228 | *∆pyrE2∆minD2* | (Nußbaum et al., 2020) |
| HTQ241 | *∆pyrE2∆minD4∆minD2* | (Nußbaum et al., 2020) |
| HTQ256 | *∆pyrE2∆flaD1∆minD2* | This study |
| HTQ255 | *∆pyrE2∆flaD1∆minD2∆minD4* | This study |
| HTQ247 | *∆pyrE2 ∆pilB3* | (Nußbaum et al., 2020) |
| HTQ248 | *∆pyrE2∆pilB3∆minD2* | (Nußbaum et al., 2020) |
| HTQ451 | *∆pyrE2∆cheW∆minD2* | This study |
| HTQ452 | *∆pyrE2∆HVO_0596* | This study |
| HTQ453 | *∆pyrE2∆cheW∆minD2∆minD4* | This study |
| HTQ454 | *∆pyrE2∆cetZ5* | This study |
| HTQ456 | *∆pyrE2∆cetZ6* | This study |
| HTQ457 | *∆pyrE2∆cetZ5∆cetZ6* | This study |
| HTQ460 | *∆pyrE2∆minD2∆HVO_0596* | This study |
| ***E.coli*** |  |  |
| 10-beta Competent Cells “TOP10” | Δ*(ara-leu) 7697 araD139 fhuA* Δ*lacX74 galK16 galE15 e14-*ϕ*80*d*lacZ*Δ*M15 recA1 relA1 endA1 nupG rpsL* (Str^R^) *rph spoT1* Δ*(mrr-hsdRMS-mcrBC)* | New England Biolabs |
| *dam^-^*/*dcm^-^*  Competent cells | *ara-14 leuB6 fhuA31 lacY1 tsx78 glnV44 galK2 galT22 mcrA dcm-6 hisG4 rfbD1 (zgb210::Tn10)* Tet^S^ *endA1 rspL136* (Str^R^) *dam13::Tn9* (Cam^R^) *xylA-5 mtl-1 thi-1 crB1 hsdR2* | New England Biolabs |

**Table 2: plasmid used in this study**

| **Plasmids** | **Description** | **Primer used** | **Enzyme used** | **Source/reference** |
| --- | --- | --- | --- | --- |
| pTA131 | Integrative plasmid with a *pyrE2* selection marker for knock-outs in *H. volcanii* (Amp^r^) | - | - | (Allers et al., 2004) |
| pTA1392 | Overexpression plasmid for *H. volcanii* under the control of a tryptophan inducible promotor. Contains *pyrE2* selection marker (Amp^r^) | - | - | (Gamble-Milner R, 2016) |
| pIDJL40 | Plasmid to express proteins with a C-terminal gfp phusion under the control of a tryptophan inducible promotor. Contains *pyrE2* selection marker (Amp^r^) | - | - | (Duggin et al., 2015) |
| pHVID21 | Plasmid to express proteins with a C-terminal mTurquoise under the control of a tryptophan inducible promotor. Contains *pyrE2* selection marker (Amp^r^) | - | - | Duggin lab |
| pSVA1841 | Integrative plasmid with a *pyrE2* selection marker to knock-out *hvo_0595* (*minD2*)(Amp^r^) | - | - | (Nußbaum et al., 2020) |
| pTQ99 | Integrative plasmid with a *pyrE2* selection marker to knock-out *hvo_1200* (flaD1/arlD1)(Amp^r^) | - | - | (Li et al., 2019) |
| pSVA5029 | Integrative plasmid with a *pyrE2* selection marker to knock-out *hvo_1225* (cheW)(Amp^r^) | - | - | (Li et al., 2019) |
| pSVA5993 | Integrative plasmid with a *pyrE2* selection marker to knock-out *hvo_0596* (Amp^r^) | 11068-11071 | - | This study |
| pSVA6037 | Integrative plasmid with a *pyrE2* selection marker to knock-out *hvo_2013 (CetZ5)* (Amp^r^) | 10651-10654 | - | This study |
| pSVA6038 | Integrative plasmid with a *pyrE2* selection marker to knock-out *hvo_2068 (CetZ6)* (Amp^r^) | 10655-10658 | - | This study |
| pSVA6039 | Integrative plasmid with a *pyrE2* selection marker for double knock-out *hvo_0595 and hvo_0596 (minD2 and HVO_0596)* (Amp^r^) | 6919,6920,  10659,10660 | - | This study |
| pSVA3919 | Plasmid to express *flaD1* with a C- terminal gfp-tag(Amp^r^) | - | - | (Li et al., 2019) |
| pSVA5031 | Expression plasmid for CheW-GFP | - | - | (Li et al., 2019) |
| pSVA3920 | Expression plasmid for MinD2 with a C-terminal GFP | 6575,6576 | NdeI, BamHI | This study |
| pSVA3926 | Expression plasmid for N-terminal GFP with MinD2 | 8009, 8010 | NheI, BamHI | This study |
| pSVA6010 | Expression plasmid for N-terminal His tag-MinD2 | 10605, 10606 | NcoI, EcoRI | This study |
| pSVA6011 | Expression plasmid for tag-less MinD2 | 6575, 10606 | NdeI, EcoRI | This study |
| pSVA6059 | Expression plasmid for a N-terminal GFP-Linker-MinD2 | 10697, 10698 | NheI,NheI | This study |
| pSVA6017 | Expression plasmid for MinD2with C-terminal HA tag for IP experiment | 10605,10610  10614,10615 | NcoI, EcoRI | This study |
| pSVA6307 | Expression plasmid for MinD2 with a C-terminal mTurquoise | 6575, 10628 | NdeI, BamHI | This study |
| pSVA6040 | Expression plasmid for N-terminally tagged HA-GFP-CetZ5 | 10665, 10666 | NheI, BamHI | This study |
| pSVA6042 | Expression plasmid for N-terminally tagged HA_GFP-CetZ6 | 10669, 10670 | NheI, BamHI | This study |
| pSVA6051 | Expression plasmid for n-terminal taggedmNeonGreen- HVO_0596 | 10647, 10648 | NheI, BamHI | This study |

**Table 3: Primer used in this study**

| **Primer number** | **Sequence** | **Description** |
| --- | --- | --- |
| 6575 | GTTCTACATATGGTCGAGGCGTTCGCCGTCGCCAG | Forward primer for MinD2 with C-terminal GFP tag with NdeI restriction site |
| 6576 | GTAGGATCCCTCGGGGACGACGGCGCTTTTG | Reverse primer for MinD2 with C-terminal GFP tag with BamHI restriction site |
| 8009 | TCTAGCTAGCGAGGCGTTCGCCGTCGCCAG | Forward primer for MinD2 with N-terminal GFP tag with NheI restriction site |
| 8010 | CTAGGATCCGGGATTCTCATATTCGCTC | Reverse primer for MinD2 with N-terminal GFP tag with BamHI restriction site |
| 10605 | AGGACCATGGTCGAGGCGTTCGC | Forward primer for MinD2 with NcoI restriction site |
| 10606 | CGCGAATTCTCATATTCGCTCG | Reverse primer for MinD2 with EcoRI restriction site |
| 10610 | TAAGCGGGAATTCTCACGCGTAGTCCGGGACGTCGTACGGGTAGCTGCCTATTCGCTCGGGGA | Forward primer to insert MinD2 for C-terminal HA tag with EcoRI  restriction site |
| 10628 | GAACGGATCCTATTCGCTCGGGGACGACGG | Forward primer to amplify minD2 with BamHI restriction site |
| 10647 | AGGTGGCTAGCAGAATCCCGCGGGGCGAA | Forward primer to insert HVO_0596 with NheI restriction site |
| 10648 | ACCTGGATCCTCAGCGACTGTCCGGGCCGGC | Reverse primer to insert HVO_0596 with BamHI restriction site |
| 10651 | GGCGAATTGGGTACCTTCCCGACGACCGACGACTG | Forward primer for US gene amplification for CetZ5 deletion-overlap PCR (orange represent overlap to plasmid pTA131) |
| 10652 | CCCGACCGCCTCGACGATAGCTCCCG | Reverse primer for US gene amplification for CetZ5 deletion- overlap PCR |
| 10653 | GTCGAGGCGGTCGGGCGTCTCTCTTTGACACGTC | Forward primer for DS gene amplification for CetZ5 deletion- overlap PCR (blue overlap US region) |
| 10654 | GGCGGCCGCTCTAGATCACTCGTCGCCCGCGCG | Reverse primer for DS gene amplification for CetZ5 deletion- overlap PCR (orange represent overlap to plasmid pTA131) |
| 10655 | GGCGAATTGGGTACCACTCAGCGGTAAATCCGATC | Forward primer for US gene amplification for CetZ6 deletion- overlap PCR (orange represent overlap to plasmid pTA131) |
| 10656 | ACGCACGGGGGTCGATAAACGTCGC | Reverse primer for US gene amplification for CetZ6 deletion- overlap PCR |
| 10657 | TCGACCCCCGTGCGTGTTGTCTGCTCTGCGACGTACC | Forward primer for DS gene amplification for CetZ5 deletion- overlap PCR (blue overlap US region) |
| 10658 | GGCGGCCGCTCTAGATCGTGCTCGCGCTCGGCGGC | Reverse primer for DS gene amplification for CetZ6 deletion- overlap PCR (orange represent overlap to plasmid pTA131) |
| 10659 | ATTACCATATGCACGCTTCTCGACGGTTGAG | Forward primer for double deletion of minD2 and HVO_0596 with NdeI restriction site |
| 10660 | ATTACCATATGCACGCTTCTCGACGGTTGAG | Reverse primer for double deletion of minD2 and HVO_0596 with XbaI restriction site |
| 10665 | ATTACCATATGCACGCTTCTCGACGGTTGAG | Forward primer to amplify CetZ5 with nheI restriction site |
| 10666 | TatgGGATCCTCAGAACAGCGAGTCGAGGC | Reverse primer for CetZ5 amlification with BamHI site |
| 10669 | TACCGCTAGCAACGTGTTCTGCTTTGG | Forward primer to amplify CetZ6 with NheI restriction site |
| 10670 | attgGGATCCTCACGCGTCGCCCGAGTCAC | Reverse primer for CetZ6 amlification with BamHI site |
| 10697 | CGCGCTAGCCTTGAGGGTAGCGGACAAGG | Forward primer to incorporate semi-flexible Linker (from Alex Bisson) |
| 10698 | CGCGCTAGCGCCTTGACCTGGGCCAGATC | Reverse primer to incorporate semi flexible Linker |
| 11068 | GGCGAATTGGGTACCACTGCGAAAGCGAACGATTG | Forward primer to amplify US gene for deletion of Hvo_0596. Overlap PCR |
| 11069 | CAACCGTCGAGAAGCGTGTCGCCCCGCGGGATTC | Reverse primer to amplify US gene for deletion of Hvo_0596. Overlap PCR |
| 11070 | GAATCCCGCGGGGCGACACGCTTCTCGACGGTTGAG | Forward primer to amplify DS gene for deletion of Hvo_0596. Overlap PCR |
| 11071 | GGCGGCCGCTCTAGACCAGTCCGCGAAGTCGG | Reverse primer to amplify DS gene for deletion of Hvo_0596. Overlap PCR |

**Table 4: Swimming motility of various mutants used in this study.** Average diameter of motility rings measured relative to the wild type, from different strains.

**Table 5: syntTax report of conserved MinD2 and HVO_0596 proteins**


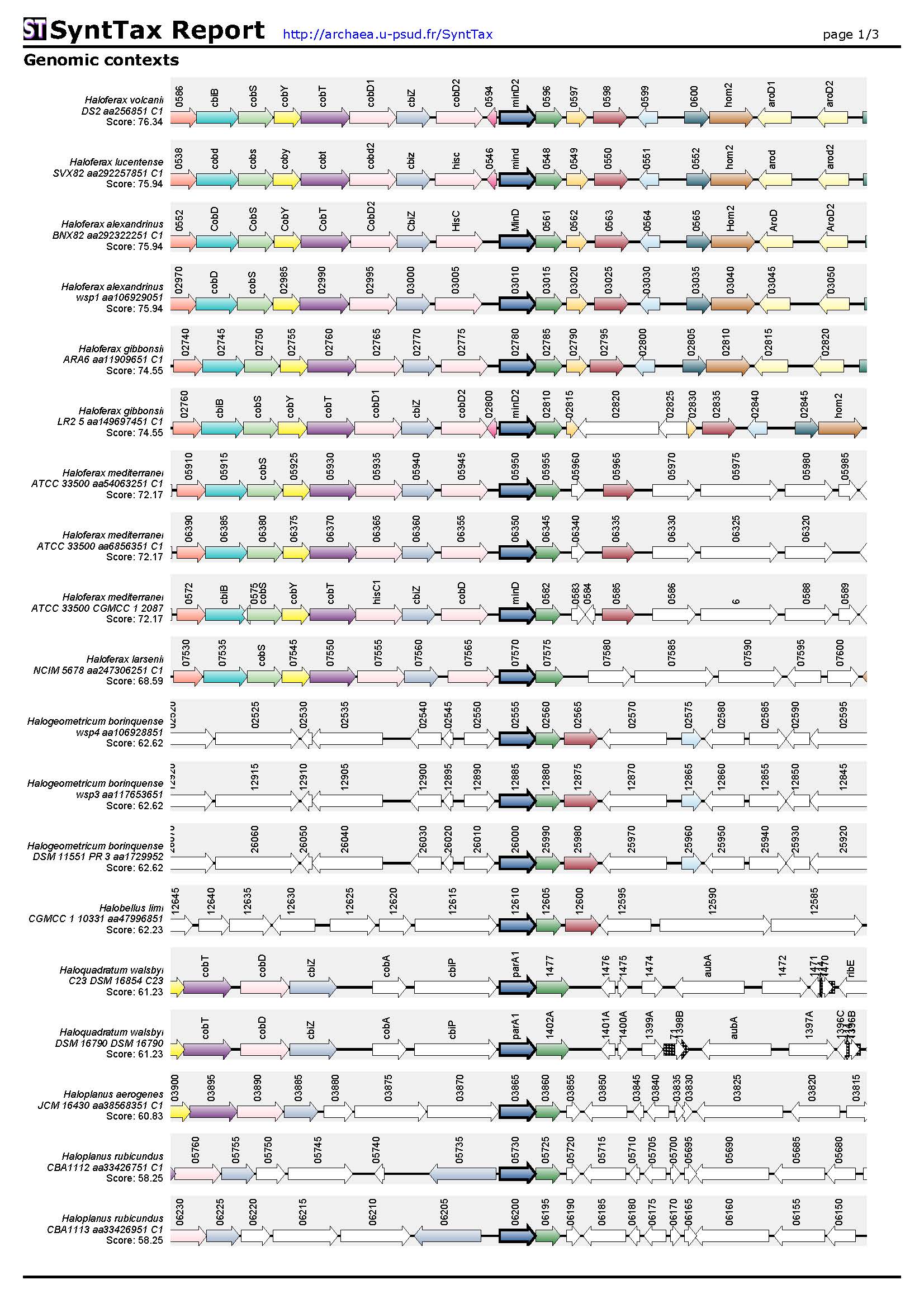


**Table 6: log_2_-fold changes
Table 7 : transcript per million values
Table 8: Functional enrichment analysis**
